# Understanding low sensitivity of community-based HIV rapid testing: experiences from the HPTN 071 (PopART) trial in Zambia and South Africa

**DOI:** 10.7448/IAS.20.7.21780

**Published:** 2017-08-29

**Authors:** Peter Bock, Comfort Phiri, Estelle Piwowar-Manning, Barry Kosloff, Nomtha Mandla, Alicia Young, Anelet James, Ab Schaap, Michelle Scheepers, Deborah Donnell, Sam Griffith, Wafaa El-Sadr, Kwame Shanaube, Nulda Beyers, Richard Hayes, Sarah Fidler, Helen Ayles, on behalf of the HPTN 071 (PopART) Study Team

**Affiliations:** ^a^ Desmond Tutu TB Centre, Department of Paediatrics and Child Health, Stellenbosch University, Western Cape, South Africa; ^b^ Zambart, University of Zambia, Lusaka, Zambia; ^c^ Department of Pathology, HPTN Laboratory Center, Johns Hopkins University School of Medicine, Baltimore, MD, USA; ^d^ Department of Clinical Research, London School of Hygiene and Tropical Medicine, London, UK; ^e^ Statistical Center for HIV/AIDS Research and Prevention, Fred Hutchinson Cancer Research Center, Seattle, WA, USA; ^f^ Department of Infectious Disease Epidemiology, London School of Hygiene and Tropical Medicine, London, UK; ^g^ Science Facilitation Department, FHI 360, Durham, NC, USA; ^h^ ICAP at Columbia University, Mailman School of Public Health, Columbia University, New York, NY, USA; ^i^ Department of Medicine, Imperial College London, London, UK

**Keywords:** HIV rapid test, community, household, sensitivity, quality control, HPTN 071 (PopART)

## Abstract

**Introduction**: Population-wide HIV testing services (HTS) must be delivered in order to achieve universal antiretroviral treatment (ART) coverage. To accurately deliver HTS at such scale, non-facility-based HIV point-of-care testing (HIV-POCT) is necessary but requires rigorous quality assurance (QA). This study assessed the performance of community-wide HTS in Zambia and South Africa (SA) as part of the HPTN 071 (PopART) study and explores the impact of quality improvement interventions on HTS performance.

**Methods**: Between 2014 and 2016, HIV-POCT was undertaken within households both as part of the randomly selected HPTN 071 research cohort (Population Cohort [PC]) and as part of the intervention provided by community HIV-care providers. HIV-POCT followed national algorithms in both countries. Consenting PC participants provided a venous blood sample in addition to being offered HIV-POCT. We compared results obtained in the PC using a laboratory-based gold standard (GS) testing algorithm and HIV-POCT. Comprehensive QA mechanisms were put in place to support the community-wide testing. Participants who were identified as having a false negative or false positive HIV rapid test were revisited and offered retesting.

**Results**: We initially observed poor sensitivity (45–54%, 95% confidence interval [CI] 31–69) of HIV-POCT in the PC in SA compared to sensitivity in Zambia for the same time period of 95.8% (95% CI 93–98). In both countries, specificity of HIV-POCT was >98%. With enhanced QA interventions and adoption of the same HIV-POCT algorithm, sensitivity in SA improved to a similar level as in Zambia.

**Conclusions**: This is one of the first reports of HIV-POCT performance during wide-scale delivery of HTS compared to a GS laboratory algorithm. HIV-POCT in a real-world setting had a lower sensitivity than anticipated. Appropriate choice of HIV-POCT algorithms, intensive training and supervision, and robust QA mechanisms are necessary to optimize HIV-POCT test performance when testing is delivered at a community level. HIV-POCT in clients who did not disclose that they were on ART may have contributed to false negative HIV-POCT results and should be the topic of future research.

## Introduction

Globally, 37 million people are estimated to be living with HIV [1]. In 2014, UNAIDS announced a global target of 90% of HIV-positive individuals knowing their HIV status in order to deliver universal access to antiretroviral treatment (ART) for all people living with HIV (PLWH) [2]. However, there remains a considerable HIV testing gap, with only 54% of PLWH aware of their HIV status in 2014 [3]. Reaching the UNAIDS 90-90-90 targets will require a massive scale-up of HIV testing and will necessitate innovative strategies to achieve this goal.

Whilst HIV testing services (HTS) are usually provided at healthcare facilities, multiple barriers prevent wide-scale access and acceptance of testing through this approach [4]. To improve knowledge of HIV status, non-facility-based HIV testing approaches have been explored [5,6] and many are now integrated into community testing programmes [5,7–9]. Previous studies have shown high levels of competency in HIV testing amongst counsellors in household settings [10,11], and high levels of acceptance for community-based HIV testing are reported [5]. However, the quality assurance (QA) of this mode of HIV testing may be more challenging. The sensitivity and specificity of HIV point-of care testing (HIV-POCT) may be affected by user training and competency, testing environments, the algorithm used, test kit handling and storage as well as test kit performance [12–14]. Sensitivity and specificity of commonly used HIV-POCT in laboratory conditions are high (consistently 97–99%) [5,15,16]. However, there are limited and varied data on the performance of HIV-POCT in field settings, and comparison to a laboratory-based gold standard (GS) is uncommon [15,16]. The World Health Organization (WHO) pre-qualifies certain HIV testing strategies [7], but countries may utilize algorithms based on price and availability of test kits.

HIV-POCT QA guidelines vary across settings. The WHO emphasizes the importance of QA supported by well-structured quality management services and has recently updated its guidance for establishing HIV testing QA. The WHO recommends using a combination of quality control (QC) of HIV test kits and monitoring of proficiency of the staff conducting tests using both internally and externally generated plasma panels [7]. Effective implementation of these guidelines is resource-intensive and requires basic equipment and laboratory infrastructure that may be difficult to access in many high-burden settings [7].

HPTN 071 (PopART) is a community-randomized trial investigating the impact of a combination HIV prevention package on HIV incidence. The design of the study has been reported previously [17]. A key component of the combination prevention package is community-wide HIV testing offered by a novel cadre of community HIV-care providers (CHiPs) within the households of consenting individuals using HIV-POCT. CHiPs workers are “lay counsellors” who have a minimum of grade 11 or 12 high school education prior to employment and received basic accredited HIV counselling and testing training prior to conducting HIV-POCT in the field. In parallel with the CHiPs HIV testing, a randomly selected research Population Cohort (PC) of participants consented to provide an annual blood sample to determine HIV status in study laboratories for the study’s primary endpoint; many of these individuals also accept optional HIV-POCT delivered by research nurses in their households. This cohort provides an opportunity to assess performance of community-wide HIV-POCT compared to a laboratory-based GS. This manuscript describes the performance of community-wide HIV-POCT in Zambia and South Africa (SA) as part of the HPTN 071 (PopART) study.

## Methods

Within each of the 21 communities in Zambia and SA included in the HPTN 071 (PopART) study, a random sample of approximately 2000 participants aged between 18 and 44 years were selected to join the PC. Consenting participants were visited in their households and asked to provide a venous sample of blood for laboratory-based HIV testing (blinded for study arm) to inform the study primary endpoint (HIV incidence). Results of this laboratory HIV testing were not routinely returned to study participants. All participants were encouraged to undergo HIV-POCT using the current nationally approved test algorithm. The results of this testing were given directly to the participant. Not all PC participants chose to have a HIV-POCT; some may already have been tested by the CHiPs or have previously known their status. For this paper, data from the baseline survey of the PC (PC0) and the 12-month follow-up survey (PC12) were analysed.

### HIV-POCT testing algorithms

In both Zambia and SA, HIV-POCT was undertaken by both trained CHiPs (lay counsellors) for the community combination prevention intervention and research nurses for the PC. In both cases, two HIV-POCT tests performed in series were used, in line with national and local guidelines. In Zambia, the Alere Determine™ HIV-1/2 test (Alere inc., CA, USA) was used for screening and the Uni-Gold HIV test (Trinity Biotech, Bray, Co.Wicklow, Ireland) was used for confirmation throughout the study period.

In SA testing followed the national algorithm which varied during the study period. From January to June 2014, the First Response™ HIV 1-2-0 Card Test (Real Relief India Private Limited, Tamil Nadu, India) was used for screening and the Alere Determine™ HIV-1/2 for confirmation; from July to December 2014, SD Bioline HIV-1/2 3.0 (Alere, CA, USA) for screening and Alere Determine™ HIV-1/2 test was used for confirmation; from January to June 2015, the ADVANCED QUALITY™ Rapid Anti-HIV (1&2) Test (InTec Products Inc., Haicang, Xiamen, China) was used for screening and the Abon HIV 1/2/O Tri-line test (Alere Inc., CA, USA) was used for confirmation. These changes in tests kits matched those of the SA Department of Health (SADOH) which provided the study with test kits during that period.

Following the analysis of the performance of these HIV-POCT algorithms, the study team chose to provide kits for SA HIV-POCT from July 2015 onwards such that Alere Determine™ HIV-1/2 test was used for screening and the Uni-Gold™ Recombigen® HIV-1/2 test was used for confirmation, to be consistent with the algorithm used in Zambia.

### HIV-POCT quality management

A system of quality management for the HIV-POCT was developed which included both QC for the test kits and QA of the testing procedure (QA/QC). This system used nationally available guidelines, but was expanded by the study team to include internal quality control (IQC) panel testing of test kits, temperature monitoring of test kits and proficiency testing of all staff conducting HIV testing. In Zambia, additional procedures were established earlier than in SA, as initially the SA test kits were provided by the DOH and QC systems used by DOH were assumed to be adequate. The timing of the implementation of additional procedures by the study team is shown in Table 1. Details of the additional procedures are as follow:

*IQC* panel testing of test kits was performed (i) when new tests kits were delivered to study head office, (ii) after transport of test kits to site offices within the communities and (iii) monthly for test kits that had been stored at site offices and transported in the field. Due to the large number of test kits used, panels used for IQC testing were generated by each in-country study laboratory. In Zambia, IQC activities described in this paper were initiated at the beginning of the study whilst in SA QC of test kits was conducted by the SADOH initially but was undertaken by the study team from Q1 2015 onwards.

Temperature monitoring during test kit storage was conducted in each country at the in-country study head office, at field offices and in cooler boxes that were used to transport HIV-POCT kits in the field. In instances where out-of-range temperatures were reported (>27°C for three consecutive days), IQC was performed for the affected test kits as described above.

User proficiency to perform the HIV-POCT kit procedures according to the manufacturers’ specifications was assessed among all PC research staff and among CHiPs. In both countries, PC research nurses and CHiPs completed regular internal and external proficiency testing (EQA).

A checklist was developed to be used for observation of all staff performing HIV-POCT. This checklist covered all aspects of home-based testing, including: preparing the testing environment, obtaining a finger stick sample, carrying out testing and interpreting results (see Appendix). In addition, in both countries, internal proficiency panel testing was done with blinded plasma panels of HIV-positive and HIV-negative samples at least once per year for all testers. EQA with samples provided by the National Health Laboratory Service (NHLS) in SA and the National Virology Reference Laboratory (NVRL) in Zambia was also conducted on an annual basis from 2015 when these panels were made available.

If an individual staff member failed internal- or external proficiency testing, the individual underwent re-training and repeat proficiency testing before being allowed to resume HIV testing.

### Laboratory-based HIV testing

In this large clinical trial, special algorithms were developed for laboratory-based HIV testing in the PC. In addition to HIV-POCT described above which was part of the study intervention, venous blood was collected from each PC study participant for laboratory-based testing to provide data for the primary study endpoint of HIV incidence. This testing was done in two stages. In the first step, a single HIV screening assay (Abbott Architect Combo) was performed in-country. The results of that test dictated the algorithm that was used at the HPTN Laboratory Center (HPTN-LC, Johns Hopkins Univ. School of Medicine, Baltimore, MD, USA) for QA and HIV confirmation. For 10% of the samples where the in-country test was non-reactive, testing was repeated at the HPTN-LC with the same 4th generation test (the Abbott Architect Combo). If the results of the two tests were discrepant, samples were tested with the 4th generation Bio-Rad HIV 1/2 Combo (Bio-Rad Combo test) and the Bio-Rad Geenius discriminatory assay. For all samples that had a reactive in-country test, testing was performed at the HPTN-LC with a different 4th generation test (the Bio-Rad 4th generation assay). If the in-country and HPTN-LC test results were discrepant, samples were tested at the HPTN-LC with Abbott Architect assay, the Bio-Rad Geenius discriminatory assay and HIV viral load testing. The final HIV status determined at the HPTN-LC is defined in this paper as the GS. Results of HIV tests performed in the in-country laboratories and at the HPTN-LC were not reported to study participants, unless discrepancies were identified between HIV-POCT among those who accepted the testing and final laboratory test results.

### Management of discrepant results between laboratory test and HIV-POCT

In both countries, PC participants who had discrepant results for the laboratory-based test and HIV-POCT were revisited by the research staff and offered the opportunity for repeat HIV testing using HIV-POCT; this was followed by collection of an additional venous blood sample in cases where the HIV-POCT was still discrepant with the laboratory result. Information was also collected regarding prior knowledge of HIV status, engagement in care if aware of HIV-positive status and ART at the time of initial HIV-POCT.

### Data management and statistical analysis

Data for all PC participants were collected electronically using a specially designed database. All participants were identified by a unique barcode. HIV-POCT results were recorded first on a barcoded paper-based results form by the nurse, and this information was entered into the electronic data capture device at the end of each day by the research assistant. All blood samples were labelled using the participant barcode and sent to laboratories for processing within 6 h of blood draw. Aliquots of plasma were stored at −80°C until laboratory testing. All laboratory data were entered into a laboratory data management system. In the case of discrepant results between laboratory test and HIV-POCT, data entry errors were excluded by retrieval of the source document HIV-POCT form and comparison and correction on the electronic data base.

This analysis of performance of HIV-POCT compared to a laboratory reference standard was limited to those PC participants with both an HIV-POCT result and a laboratory HIV test result corresponding to PC visits taking place between January 2014 and June 2016. Estimates of sensitivity and specificity of HIV-POCT over time, with exact binomial 95% confidence intervals (95% CI), were calculated in order to assess the possible effects of test kit choice and improvement in quality management.

### Ethical approval

Ethical approval for the HPTN 071 study was obtained from the University of Zambia research ethics committee, Stellenbosch University health research ethics committee and the London School of Hygiene and Tropical Medicine ethics committee.

## Results

### Study population

Data analysed in this paper include 21,668 paired HIV-POCT and laboratory GS results obtained from 17,680 PC participants at the PC enrolment and/or 12-month follow-up surveys (16,280, 75.1% Zambia, 5388, 24.9% SA).

### HIV-POCT performance

**Figure 1.**
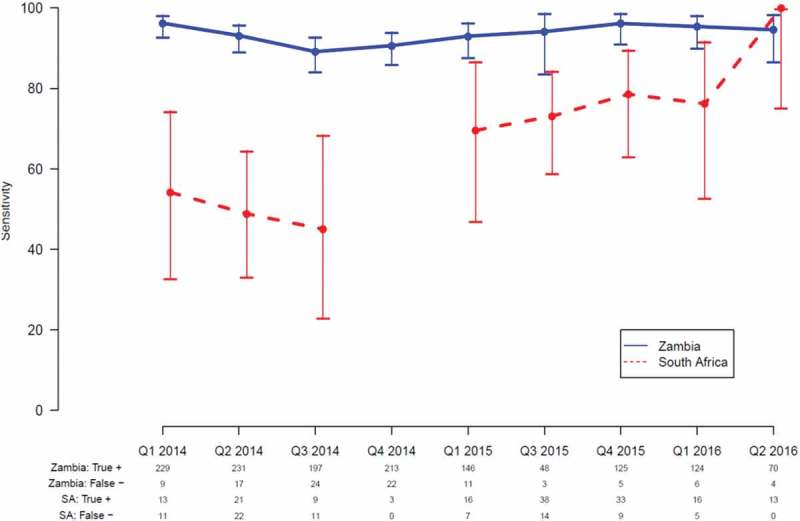
Sensitivity of HIV-POCT in Zambia and South Africa by quarter. HIV-POCT: HIV point-of-care testing.

Using data from PC participants who had both HIV-POCT and laboratory results available, we examined HIV-POCT performance over time by quarter. Figure 1 summarizes HIV-POCT sensitivity for each country. Table 1 shows sensitivity and specificity by country over time alongside the test kit algorithms and other quality management activities.

**Table 1.** Performance, test kits used and quality measures in Zambia (Z) and South Africa (SA)

	Q1 2014	Q2 2014	Q3 2014	Q4 2014	Q1 2015	Q2 2015	Q3 2015	Q4 2015	Q1 2016	Q2 2016
Zambia										
*N* (total test)	1317	2038	2346	2318	2103	0^a^	822	2002	2194	1140
Correctly identified HIV-positive (HIV-POCT+/GS +)	229/238	231/248	197/221	213/235	146/157		48/51	125/130	124/130	70/74
Correctly identified HIV-negative (HIV-POCT−/GS−)	1077/1079	1788/1790	2121/2125	2081/2083	1944/1946		767/771	1871/1872	2063/2064	1065/1066
Sensitivity % (95% CI)	96.2 (93–98)	93.1 (89–96)	89.1 (84–93)	90.6 (86–94)	93.0 (88–96)		94.1 (84–99)	89.9 (84–94)	95.4 (90–98)	94.6 (87–98)
Specificity % (95% CI)	99.8 (99.3–100)	99.9 (99.6–100)	99.8 (99.5–100)	99.9 (99.7–100)	99.9 (99.6–100)		99.5 (98.6–100)	99.9 (99.6–100)	100 (99.7–100)	99.9 (99.5–100)
Zambia first-line POCT	Determine	Determine	Determine	Determine	Determine		Determine	Determine	Determine	Determine
Zambia second-line POCT	Uni-Gold	Uni-Gold	Uni-Gold	Uni-Gold	Uni-Gold		Uni-Gold	Uni-Gold	Uni-Gold	Uni-Gold
IQC test strips/devices (pass/tested)	67/67	95/95	615/615	752/752	423/423	1164/1164	2840/2840	2874/2874	3543/3543	2528/2528
Panel proficiency testing (pass/total)		96/99	79/82	143/151	98/100l	108/110	141/144	120/124	55/55	102/106
South Africa										
*N* (total test)	429	672	395	90	453	0^a^	1029	911	973	436
Correctly identified HIV-positive (HIV-POCT +/GS +)	13/24	21/43	9/20	3/3	16/23		38/52	33/42	16/21	13/13
Correctly identified HIV-negative (HIV-POCT−/GS−)	405/405	629/629	375/375	87/87	430/430		977/977	868/869	952/952	423/423
Sensitivity % (95% CI)	54.2 (33–74)	48.8 (33–65)	45.0 (23–68)	^b^	69.6 (47–87)		73.1 (59–84)	78.6 (63–90)	76.2 (53–92)	100 (75–100)
Specificity % (95%CI)	100 (99–100)	100 (99–100)	100 (99–100)	100 (96–100)	100 (99–100)		100 (99.6, 100)	99.9 (99–100)	100 (99.6, 100)	100 (99–100)
SA first-line POCT	First response	First response	SD Bioline	SD Bioline	Advance quality		Advance quality	Determine	Determine	Determine
SA second-line POCT	Determine	Determine	Determine	Determine	Abon		Abon	Uni-Gold	Uni-Gold	Uni-Gold
IQC test strips/devices (pass/tested)					1482/1482	556/556	718/718	2297/2297	2131/2131	3090/3090
Panel proficiency testing (pass/total)					4/4	119/122	129/130	43/43	32/34	0

HIV-POCT+: final result of HIV-POCT algorithm is positive; HIV-POCT−: final result of HIV-POCT algorithm is negative; GS+: final result of laboratory algorithm is positive; GS−: final result of laboratory algorithm is negative. HIV-POCT: HIV point-of-care testing; IQC: internal quality control; GS: gold standard; QA: quality assurance; PC: Population Cohort. ^a^No PC activity this quarter but QA continued. ^b^Sensitivity not calculated due to small number of positive results.

Data from Zambia for the entire period showed a sensitivity of 89–96%, with the lower limit of the 95% CI remaining above 84% throughout. However, the sensitivity of HIV-POCT in SA was very different, with observed sensitivity as low as 45%.

In SA the test kit algorithm changed first in Q3 2014 in line with SA national guideline change and again in Q1 2015. Neither of these changes in HIV-POCT algorithm appeared to significantly change the performance of the testing process. As a consequence of continuing poor performance in SA, HIV-POCT algorithm was changed in Q4 2015 to be consistent with that used in Zambia (Alere Determine™ HIV-1/2 followed by Uni-Gold™ Recombigen® HIV-1/2). Additional quality management procedures were also employed to monitor HIV-POCT performance, similar to what was being implemented in Zambia. These included re-training of all staff and more frequent staff supervision. Proficiency testing using approved plasma panels was introduced.

### Quality assurance

IQC testing was performed on a total of 25,175 test strips/devices overall at central storage and field sites, as well as when temperature monitoring showed deviations from the recommended storage temperatures in storage sites or field cooler boxes. On all occasions, the test strips/devices tested, passed (IQC) (Table 1).

Internal proficiency panel testing was conducted annually so that during this period individual testers may have been tested more than once. A total of 971 proficiency panels were used (934 for CHiPs and 37 for PC nurses) in Zambia with an overall pass rate of 96% (Table 1). External proficiency panel testing was conducted once during the period of this report and 419/444 testers (94%) passed (20 PC nurses were tested with 100% pass rate). In SA, internal proficiency panel testing started later and a total of 333 proficiency panels being used (271 for CHiPs and 62 for PC nurses) with an overall pass rate of 98%. All individuals failing proficiency panel testing were re-trained and had to pass a further proficiency panel test before being allowed to resume testing. External panel proficiency testing was conducted in the six HPTN 071 intervention sites with one panel per site being tested rather than individual testers. All six sites were tested on four occasions with one site failing on one occasion. This site received additional re-training.

Observation of all steps in the HIV-POCT process using the supervision checklist started in 2015, and observations using this revealed that most errors were made in the finger stick and correct use of the sample collection device (capillary tube or pipette according to test used). Errors were also made in the timing and amount of chase buffer added.

### Follow-up of individuals with discrepant HIV-POCT and laboratory tests

Overall, 199 participants had 200 discrepant HIV results (participants were seen annually so it was possible for them to receive discrepant results in both years). Figure 2 summarizes for each country the follow-up of participants with test results that were discrepant between the HIV-POCT and the laboratory GS. In Zambia 120 and in SA 80 participants were identified with discrepant results. Multiple attempts to revisit all these participants were made by the research teams in both countries, according to a standardized algorithm, during which these participants were offered a repeat HIV-POCT and laboratory test. There were some differences in the procedures for conducting re-test visits between Zambia and SA.

**Figure 2a.**
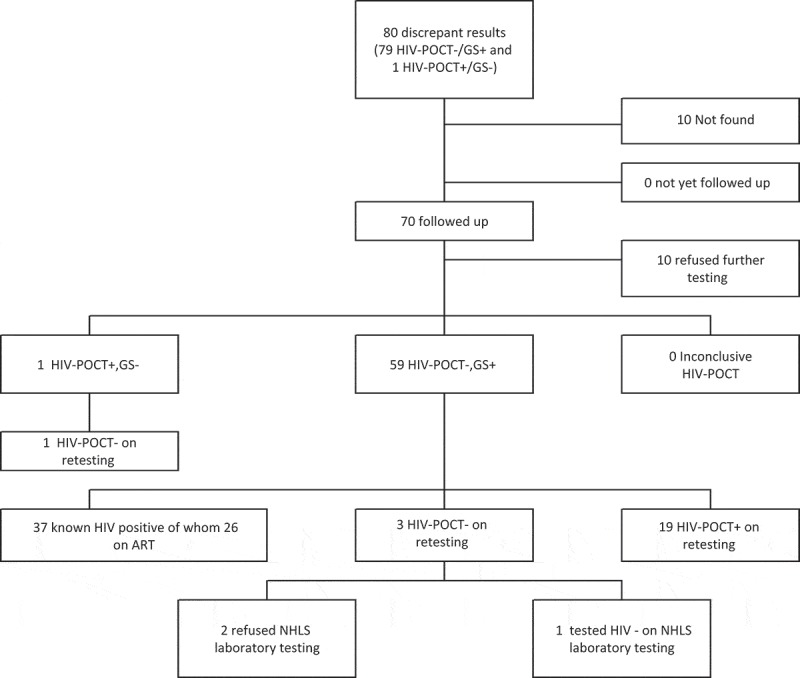
Flow chart of follow up of participants with discrepant HIV results South Africa. HIV-POCT: HIV point-of-care testing. HIV-POCT−: original HIV-POCT algorithm negative; HIV-POCT+: original HIV-POCT algorithm positive; inconclusive HIV-POCT−: original HIV-POCT algorithm discordant; GS+: laboratory algorithm (gold standard) HIV positive; GS−: laboratory algorithm negative; GS confirmed: after retesting the HIV-POCT agreed with the laboratory gold standard; HIV-POCT confirmed: after retesting the results of the repeat HIV-POCT algorithm agreed with the original HIV-POCT algorithm.

**Figure 2b.**
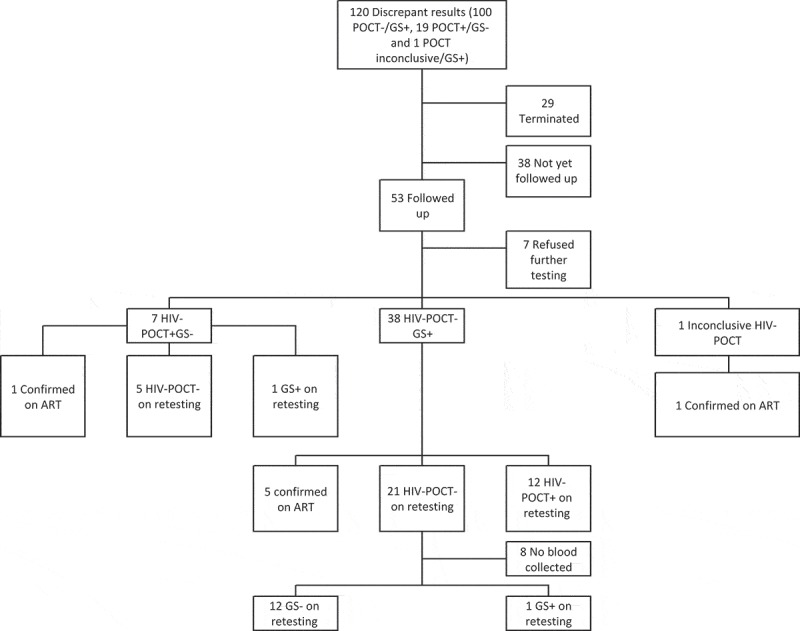
Flow chart of follow up of participants with discrepant HIV results Zambia.

In SA, re-test visits have been attempted for all 80 participants with confirmed discrepant results. PC staff were unable to locate 10 participants, and a further 10 declined a re-test visit, for the remaining 60 participants, 59 appeared to have initial false negative results (HIV-POCT-negative but GS-positive) and 1 an initial false positive result (HIV-POCT-positive but GS-negative). Of the 59 individuals with false negative results, 37 (63%) were found to already know their HIV-positive status and 26 (44%) were confirmed to be on ART at the time of the false negative POCT. Re-testing was not performed on known HIV-positives; however, they were given adherence counselling and advised to attend the clinic. For the remaining 22 individuals, HIV-POCT was repeated using the algorithm of Alere Determine™ HIV-1/2 and Uni-Gold™ Recombigen® HIV-1/2. Three of these participants again tested HIV-negative on HIV-POCT. Of these, two did not consent to further blood draw for plasma HIV testing and one tested HIV-negative on further in-country laboratory testing. Investigation of this participant was terminated after the participant was lost to follow-up due to relocation out of the study area. Including individuals known to be HIV–positive, a total of 56/59 (95%) were confirmed to have been prior false negative HIV-POCT results. One participant had a false positive HIV rapid test; this participant was re-visited and on re-testing with HIV-POCT tested HIV-negative.

In Zambia, the picture was different. Of the 120 participants with discrepant results, 29 terminated participation at a subsequent PC visit (moved out, not found or refused further participation). Due to delays in laboratory results and receipt of source data from remote sites, the follow-up results of a further 38 participants could not be included. Of the remaining 53 participants followed up, 7 participants declined further testing, leaving 46 of whom 38 initially appeared to have false negative HIV-POCT results (HIV-POCT-negative but GS-positive), 7 false positive results (HIV-POCT-positive but GS-negative) and 1 an inconclusive HIV-POCT result (discordant results between the two rapid tests used as the HIV-POCT algorithm, GS-positive). Of the 38 individuals with false negative results, 5 (13%) were already known to be HIV-positive and taking ART. The majority, 21 (55%), had repeat HIV-POCT results consistent with the original negative HIV-POCT, demonstrating some inherent differences between the laboratory and HIV-POCT and some possible laboratory errors. For the remaining 12 (32%), repeat HIV-POCT confirmed the positive laboratory result. For five out of seven apparent false positives, the repeat HIV-POCT was negative, the other two participants were confirmed to be HIV-positive, one participant confirmed that they were on ART and for the other repeat HIV-POCT and laboratory testing confirmed a positive result. Finally, the participant with an inconclusive HIV-POCT stated they were on ART at the follow-up visit.

## Discussion

Expanding high-quality community-based HIV-POCT is critical if high burden communities are to achieve the UNAIDS 90-90-90 targets. The HPTN 071 (PopART) study offered a unique opportunity to assess the performance of HIV-POCT conducted in the homes of over 17,000 participants in urban and peri-urban high HIV-burden communities in Zambia and SA. Through comparison of results from field (household) HIV-POCT testing with laboratory-based testing on venous blood samples, we noted that despite careful and repeated user training and assessment and monitoring of cold chain storage of HIV-POCT kits, the sensitivity of field HIV-POCT is less than that reported for laboratory-based HIV testing [16].

The situation in the SA sites demonstrated a “perfect storm” of poor choice of HIV-POCT algorithms, inadequate QA and user error. It is impossible to identify which contributed most to the poor performance. The requirement for staff re-training to accommodate frequent changes in the type of HIV-POCT kits procured by SADOH is likely to have contributed to user error in this setting. Change in HIV-POCT kits to consistent use of a well-established algorithm in combination with strengthened training, supervision and quality management all played a part in improving the performance.

One critical stage in the performance of HIV-POCT is sample collection. This involves the use of different manufacturer-provided sample collection tools some of which are challenging for non-laboratory staff to use, for example, the capillary tube device. Additionally, some manufacturers offer complete kits but also sell the components individually which may result in HIV-POCT being conducted without the correct sample collection device. Panel proficiency testing does not test this step and whilst the use of dried samples, as is currently recommended by WHO for QA, allows for easier shipment of QA materials, it requires different skills in rehydration and testing which do not reflect the real-life situation [7]. In the proficiency panel testing for this study with over 700 nurses and lay counsellors, the pass rate was consistently high (>95%), but user errors were detected when we implemented our increased supervision and use of a checklist (Appendix) which ensures that testers are assessed for proficiency in all stages of testing, including sample collection as well as counselling.

IQC of test kits after exposure to out-of-range temperatures in both countries did not reveal any functional abnormalities, suggesting that in this study, this factor did not contribute to the observed poor test kit performance. The number of test kits tested during internal QA was very large necessitating large quantities of positive and negative controls to be produced at a significant cost.

The laboratory GS used in this study included combined antigen–antibody 4th generation tests and viral load testing and so 3rd generation HIV-POCT will never be able to perform as well. However, it is unlikely that even with the anticipated differences in sensitivity between HIV-POCT 3rd generation antibody testing and laboratory testing, failure to identify acute infection was the primary driver of decreased sensitivity. Accounting for missed acute infections, which can be assumed to account for only a small proportion of the observed false negative HIV-POCT results, the performance of community-wide HIV-POCT was still not ideal. Laboratory testing, which was conducted during this study, is extremely labour-intensive and time-consuming and so it is not being recommended as an alternative to HIV-POCT. There is, however, a need to balance the widespread scale-up of HTS with quality of the results. Our results from the re-visits to participants with discrepant results in Zambia also show that laboratory testing may also have errors, possibly due to sample mislabelling.

The finding of increased false negative results in those individuals taking ART warrants further investigation. There is a paucity of evidence for decreased sensitivity of POCT in HIV-positive clients who are taking ART in the adult population; however, there is emerging evidence of this in children and adolescents [18,19]. HIV-POCT was not intended for use among individuals on ART, and this was an unexpected scenario in our study. In a “real-world” setting, this is a potentially important finding which requires further research and emphasizes the importance of appropriate messaging when offering community-based HIV testing, particularly with reference to limitation of HIV-POCT for individuals on ART. Further investigation of the association between ART exposure and false negative results and the possible immunological mechanisms underpinning this effect are outside the scope of this paper but should be a priority.

Few studies have been conducted comparing HIV-POCT using finger stick whole blood in field conditions with a laboratory GS. Specificity in data from the current study was high; we found very low levels of false positive rapid test results, in contrast to some studies [20]. Published data on sensitivity of HIV rapid tests in the field vary. One study from SA nested within the *Good Start Trial* showed sensitivity of 98% when comparing HIV-POCT tests with laboratory-based HIV tests [10], whereas another South African study measured accuracy of HIV-POCT testing in a clinic setting and found high rates of false negative HIV tests (sensitivity 69%, 95% CI: 41–89%) which was improved by introduction of a different testing algorithm and QA measures [13]. The authors concluded that user error was the most significant contributor to inaccuracy.

Throughout the study period, the same HIV-POCT kits and QA/QC procedures were used for the CHiPs intervention as in the PC research cohort. Whilst parallel laboratory testing was not undertaken for the community members tested by CHiPs, we assume that similar challenges of HIV-POCT sensitivity are likely to have occurred in that context. Thus, it was critical to communicate the observed poor HIV-POCT performance to the community. Throughout the conduct of the HPTN 071 (PopART) study, the study team reported the findings of HIV-POCT performance to in-country ethics committees, study communities and international advisory boards, the study sponsor and Department of Health partners. In partnership with all stakeholders, community messaging was developed and delivered. This messaging focused on encouragement of repeat HIV testing for all at-risk individuals to avoid missed HIV diagnoses and consequently compromising individual health as well as risk of onward transmission and included reference to the fact that HIV rapid tests, like other diagnostic tests, are not 100% accurate.

### Strengths and limitations

This study was conducted in the real-world setting using HIV-POCT as used in national algorithms and nationally approved QA procedures. The study setting offered a unique opportunity to compare HIV-POCT results to laboratory-based 4th generation testing completed in parallel on the same individuals. The study does, however, have limitations. It is difficult to attribute improvements in HIV-POCT sensitivity to specific factors, as multiple components of QA intervention were implemented concurrently with changes in test kits in SA. However, this is exactly how these changes would be implemented by national health systems. In the data shown here, the testing was conducted by nurses and we have assumed that similar results were seen in the HIV-POCT being done by lay counsellors at the same time using the same test algorithms and QA systems.

## Conclusions

In conclusion, this is one of the first reports of wide-scale delivery of HIV-POCT in high-burden real-world settings compared to a laboratory GS. In this study, we demonstrate that detection of HIV infection can be improved significantly with enhanced user training, implementation of frequent and vigilant QA and QC monitoring and consistent use of an approved HIV-POCT algorithm. HIV RNA testing is more sensitive for detecting HIV infection than 4th generation assays but may not be feasible or affordable in some settings.

In order to reach our goals of universal knowledge of HIV status using large-scale non-facility-based HIV testing programmes, appropriate QA procedures must be carefully established and users must be adequately trained and supervised in conducting all testing procedures. Programmes should also pay specific attention to advances in HIV-POCT technology and new evidence evaluating HIV-POCT in field settings, ensuring that they are using the best option for their setting.

## Notes

To access the supplementary material to this article please see Supplementary Files under Article Tools online.
